# Cognitive Performance and Adherence to Treatment in Patients with Obstructive Sleep Apnea

**DOI:** 10.1055/s-0046-1827497

**Published:** 2026-10-09

**Authors:** Fernando Alberto Ceballos-Fuentes, Sanket Meghpara, Rijesh Niraula, Lourdes M. DelRosso

**Affiliations:** 1 Neurology and Sleep Center, Guatemala City, Guatemala.; 2 Department of Medicine, University of California, San Francisco, Fresno, California, United States.

**Keywords:** obstructive sleep apnea, continuous positive airway pressure (CPAP), treatment adherence, Montreal cognitive assessment (MoCA), attention, residual apnea–hypopnea index

## Abstract

**Objective:**

Cognitive impairment is common in obstructive sleep apnea (OSA), but how cognition relates to positive airway pressure (PAP) adherence among asymptomatic referrals is less clear. We evaluated whether global cognition using the Montreal Cognitive Assessment (MoCA) is associated with continuous PAP (CPAP) adherence.

**Materials and Methods:**

In total, 29 OSA patients underwent the MoCA test. Data on CPAP adherence was obtained at 3 months. The Pearson correlations quantified associations between MoCA scores and adherence/effectiveness measures.

**Results:**

The mean age of the participants was of 64.3 ± 8.0 years, and 51.7% of them had MoCA scores < 26. Among those with mild OSA, who scored 5 to 15 on the apnea–hypopnea index (AHI), 57.1% were impaired despite being asymptomatic by history. Higher MoCA correlated with higher CPAP use (r = 0.65, *p* = 0.023) and showed a positive trend with nights used > 4 hours (r = 0.54, *p* = 0.073). The attention domain correlated with both average hours/day and percent nights > 4 hours, while lower language scores correlated with higher residual AHI (r = −0.69, *p* = 0.019).

**Discussion:**

The high prevalence of cognitive impairment despite the absence of self-reported symptoms suggests that cognitive deficits may be underrecognized in patients undergoing evaluation for OSA. The association of global cognition and attention with CPAP use further suggests that cognitive function may influence patients' ability to successfully engage with treatment.

**Conclusion:**

Cognitive impairment was present in 51.7% of this asymptomatic cohort and in 57.1% of participants with mild OSA. Higher global MoCA and attention scores were associated with greater CPAP use, while lower language scores were associated with higher residual AHI.

## Introduction

The National Sleep Foundation reported that approximately 90 million Americans experience isolated snoring, of whom 37 million experience it routinely. This symptom results from the abnormal vibration of soft tissue in the upper airway during sleep. Its incidence varies widely (2–86%), likely due to the lack of universal operational criteria. 1 Although it can occur without obstructive sleep apnea (OSA), being known as primary snoring, 2 it is critically important to distinguish it from OSA using polysomnography (PSG), 1 3 given the high prevalence of OSA among habitual snorers. Moreover, the consequences extend beyond the affected patient, as snoring also fragments the sleep of bed partners. 4

The OSA is a highly prevalent sleep disorder, 5 affecting 9 to 38% of middle-aged adults, 3 and rising to 88 to 90% among individuals older than 65 years. 6 Other major risk factors include male sex and obesity. 3 As a sleep-related breathing disorder, OSA is characterized by partial (hypopnea) or complete (apnea) obstruction of airflow in the upper airway, accompanied by increased respiratory effort. 7 This results in intermittent hypoxemia and hypercapnia, leading to oxidative stress, production of reactive oxygen species, activation of inflammatory cascades, and elevated proinflammatory cytokines, including interleukin (IL)-2 and IL-4, tumor necrosis factor (TNF), and C-reactive protein (CRP). These processes may induce endothelial dysfunction, threatening cardiac and cerebrovascular vasculature. Also, OSA is associated with metabolic disorders such as type 2 diabetes and poorly controlled dyslipidemia. 8

The apnea–hypopnea index (AHI) remains the primary diagnostic criterion, yet clinical practice guidelines typically differentiate between severity thresholds and the presence of symptoms when establishing a diagnosis. When the AHI exceeds 15 events/hour, OSA is diagnosed regardless of symptomatology. When the index is between 5 and 15 events/hour, the American Academy of Sleep Medicine recommends diagnosing OSA only if symptoms are present, such as excessive daytime sleepiness, cognitive impairment, mood disturbances, or the presence of cardiovascular or psychiatric disorders. 9 Despite this recommendation, cognitive assessment is not routinely performed in sleep clinics, meaning that patients with an AHI between 5 and 15 could be excluded from treatment pathways unless other risk factors exist. This phenomenon is also seen in women, who are often considered less vulnerable than men, despite evidence that cognitive decline also occurs in early stages of life in both sexes. 10 Subtle neurocognitive deficits may, therefore, go undetected when diagnostic evaluations rely solely on overt symptom reporting.

Mild cognitive impairment (MCI) is defined as the transitional stage between normal aging and dementia. Its onset can already be observed in midlife (approximately age 45), yet the underrecognition of subtle cognitive changes results in delayed diagnosis and care in 27 to 81% of affected patients. 11 Numerous studies demonstrate a robust and verifiable association between OSA and MCI, which frequently precedes the development of vascular and, less commonly, mixed dementia—vascular dementia coexisting with Alzheimer's disease, reported in 22% of the cases. 12

In a cross-sectional study 13 of 104 male patients (56 with OSA and 48 controls) who presented with snoring between 2021 and 2023, several tests were performed, including PSG, cognitive function testing with the Montreal Cognitive Assessment (MoCA) and Cambridge Neuropsychological Test Automated Battery (CANTAB), and brain magnetic resonance imaging (MRI) with Fazekas scoring for white matter hyperintensities. Results showed that patients with OSA had significantly lower MoCA scores, particularly in visuospatial, executive, and delayed recall domains, compared with controls, as well as higher Fazekas scores. 13

Another cohort 14 of 81 snoring patients underwent PSG between 2012 and 2013 and were divided into four groups according to AHI and primary snoring. Cognitive evaluation was conducted using MoCA and the Mini-Mental State Examination (MMSE). Results demonstrated progressively declining MoCA scores, with greater hippocampal atrophy and periventricular hyperintensities as OSA severity increased, 14 in addition to the substantial economic burden of the disease. 11

Furthermore, a study 15 of 203 individuals with snoring, monitored with PSG between 2016 and 2017, divided patients into no OSA, as well as mild, moderate, and severe OSA groups. All were assessed with MoCA, MMSE, and the Epworth sleepiness scale (ESS). Patients with severe OSA scored the lowest on MoCA, and younger patients exhibited impaired attention and short-term memory function. 15

In another study, 16 139 patients monitored with PSG between 2011 and 2013 were divided into four groups based on AHI and primary snoring. The MoCA, MMSE, and ESS tests were administered, and serum levels of high-sensitivity CRP (Hs-CRP), leptin, and TNF-α were measured to evaluate systemic inflammation. Questionnaire results, inflammatory markers, and PSG parameters were compared across groups, revealing that systemic inflammation and cognitive dysfunction were more common in OSA patients, with MoCA scores significantly correlated with TNF-α levels and nocturnal intermittent hypoxemia. 16

In a cross-sectional study, 17 patients were divided into no OSA, as well as mild, moderate, and severe OSA groups. Cognition was assessed using MoCA, which was also used for memory index score (MIS), and the color trail test (CTT). Patients with moderate-to-severe OSA had a higher proportion of objective cognitive impairment (64.1%), but they reported fewer subjective cognitive complaints (38.3%) compared with mild or no OSA. 17

Other studies have identified small-vessel cerebrovascular disease as a mechanism linking OSA-related hypoxemia with medial temporal lobe degeneration in older adults. 18 Additional cross-sectional analyses combining PSG and brain MRI suggest an association between OSA and white matter hyperintensities, 19 while further findings indicate that both the degree and frequency of desaturations in this condition are associated with cortical thinning in frontal and parietal regions. 20

The MoCA is a brief, 30-point screening tool administered in approximately 10 minutes. 21 It evaluates multiple cognitive domains, including attention, visuospatial/executive function, language, abstraction, memory (delayed recall), and orientation. It was specifically designed to detect subtle deficits often missed by the MMSE, particularly those related to MCI and early Alzheimer's disease. 22 In its original validation study, the MoCA demonstrated superior diagnostic accuracy compared with the MMSE in detecting both MCI and early Alzheimer's disease. 22 Cognitive impairment is a growing public health challenge in aging populations with increasing comorbidities. It is estimated that 35.6 million people worldwide were living with dementia in 2010, and that this number will nearly double every 20 years, reaching 65.7 million in 2030 and 115.4 million in 2050. 23 Recent systematic reviews and meta-analyses have further reinforced the link between OSA and cognitive dysfunction across multiple domains, including attention, executive function, and memory. 24 25 Furthermore, treatment of OSA has also shown significant improvement in neurocognitive function. 26

In the present study, we evaluated a cohort of adults referred for sleep assessment who did not report cognitive or mood symptoms, had no history of dementia or depression, were not taking psychoactive medications, and had no significant comorbidities. All participants reported snoring but were otherwise asymptomatic according to clinical history.

Cognitive function was objectively assessed using the MoCA, which measures global cognition through 10 subtests. Visuospatial abilities were evaluated with a clock-drawing task and a three-dimensional (3D) cube copy, short-term memory with two 5-word learning trials followed by delayed recall, and executive function with an adapted Trail Making B task, a phonemic fluency task, and a two-item verbal abstraction task. Attention, concentration, and working memory were assessed with a vigilance task, serial subtraction, and forward and backward digit span. Language was tested through low-familiarity animal naming (lion, camel, rhinoceros), repetition of two syntactically complex sentences, and a fluency task. Orientation was assessed by time and place. 23

Specifically, we examined whether patients with AHI values between 5 and 15, who require symptoms for an OSA diagnosis, already demonstrated measurable cognitive impairment using the MoCA psychometric criteria. 11

## Aims

1. The primary aim of this study was to determine the prevalence of cognitive impairment (MoCA score < 26) in asymptomatic individuals undergoing PSG, stratified by AHI category (< 5, 5–15, > 15). Furthermore, we aimed to identify which cognitive domains are most affected in this population and whether sex or age differences exist. Finally, we aimed to examine associations between MoCA scores and objective sleep parameters, including total sleep time, sleep efficiency, wake after sleep onset, and apnea–hypopnea indices.

## Materials and Methods

### Study Design and Population

We conducted a retrospective analysis of consecutively evaluated adult patients referred to the sleep disorders center for PSG between July 1st, 2024, and December 31st, 2024. Patients were eligible for inclusion if they were aged ≥ 18 years; reported habitual snoring; had no history of dementia or depression; were not currently taking psychoactive medications; had no significant comorbidities (e.g., stroke, severe cardiopulmonary disease); and were considered asymptomatic by clinical interview, with no self-reported memory complaints, daytime sleepiness, or other cognitive or mood symptoms.

Exclusion criteria were patients already known OSA or had previous treatment for OSA (either CPAP, mandibular advancement device, surgery, or other), recent hospitalization (within 3 months), significant uncorrected vision or hearing that can impact the test, and non-English speakers.

A formal screening log was not maintained; therefore, the total number of patients assessed for eligibility could not be determined. Participants were recruited from a private sleep center and were predominantly from a middle socioeconomic background with at least a high school level of education.

### Sleep Study and OSA Classification

Overnight PSG was performed according to the American Academy of Sleep Medicine (AASM) standards. The AHI was calculated as the total number of events per hour of sleep using standard criteria. Participants were stratified into three AHI categories: < 5 events/hour (normal range); 5 to 15 events/hour (mild OSA; diagnosis requires symptoms under current criteria); > 15 events/hour (moderate-to-severe OSA; diagnosis can be made regardless of symptoms).

Additional PSG parameters collected included total sleep time (TST), sleep latency, sleep efficiency (SE), wake after sleep onset (WASO), and time spent in each sleep stage.

### Cognitive Assessment

Cognitive function was assessed on the same day or within one week of PSG using the MoCA, which evaluates multiple cognitive domains, including visuospatial/executive function, naming, attention, language, abstraction, delayed recall, and orientation. It has been validated with internal consistency yielding a Cronbach α on the standardized items of 0.83. 21 The total score ranges from 0 to 30, with scores < 26 considered indicative of cognitive impairment.

Domain scores were also recorded, including visuospatial/executive (maximum of 5 points), naming (maximum of 3), attention (maximum of 6), language (maximum of 3), abstraction (maximum of 2), delayed recall (maximum of 5), and orientation (maximum of 6) domains.

All cognitive testing was administered by a sleep medicine specialist who had completed the official MoCA online training and certification program, ensuring standardized administration and scoring in accordance with test guidelines.

### Statistical Analysis

All analyses were conducted using IBM SPSS Statistics for Windows (IBM Corp.) software, version 29.0.

Continuous variables were expressed as mean ± standard deviation (SD) or median and interquartile range (IQR) values, as appropriate. Categorical variables were expressed as counts and percentages.

Comparisons of MoCA scores between AHI categories were performed using independent-sample *t* -tests for two groups or ANOVA for three-group comparisons. The prevalence of MoCA-defined cognitive impairment (< 26) was compared between groups using the chi-squared or Fisher's exact tests, as appropriate.

Pearson's correlation coefficients were calculated to explore associations between total MoCA score and objective sleep parameters (TST, sleep latency, SE, WASO, AHI). Age and sex differences in MoCA scores were assessed with correlation analysis (for age) and *t* -tests (for sex). Pearson correlation coefficients were calculated to examine associations between MOCA total and subdomain scores with CPAP compliance measures. Statistical significance was set at *p* < 0.05, and results are presented with correlation coefficients, *p* -values, and sample sizes.

Assumption testing indicated deviations from normality for several continuous variables based on the Shapiro–Wilk test ( *p* < 0.05), while homogeneity of variance was preserved (Levene's test *p* > 0.05) for group comparisons. Given these findings, sensitivity analyses using Spearman rank correlations were performed. These analyses demonstrated consistent direction and similar effect sizes compared with the primary Pearson correlations, with moderate positive associations between global cognition and CPAP adherence, as well as between attention and adherence, and a moderate inverse association between language performance and residual AHI, supporting the robustness of the observed relationships.

## Results

A total of 29 asymptomatic adult participants (mean age: 64.3 ± 8.0 years, 51–84) were included in the analysis. The cohort comprised 16 men (55.2%) and 13 women (44.8%). The mean total MoCA score was 25.2 ± 2.3 (18–29). Using the standard cutoff of < 26 to indicate cognitive impairment, 15 participants (51.7%) were classified as impaired.

### MoCA Domain Performance

The mean domain scores were visuospatial/executive: 4.59 (maximum score of 5; 27.6% patients scored below the maximum); naming: 3.00 (maximum of 3; 0% scored below); attention: 5.31 (maximum of 6; 44.8% scored below); language: 2.69 (maximum of 3; 31.0% scored below); abstraction: 1.90 (maximum of 2; 10.3% scored below); delayed recall: 1.59 (maximum of 5; 100% scored below); and orientation: 6.00 (maximum of 6; 0% scored below).

The most affected domain was delayed recall, with all participants scoring below the maximum, followed by attention and language.

When participants were stratified into three age groups (< 60, 60–69, and ≥ 70 years), a progressive increase in the prevalence of cognitive impairment was observed. In the < 60 group, cognitive impairment (MoCA < 26) was present in 33.3% of individuals. This proportion increased to 50.0% in the 60 to 69 age group and reached 71.4% among participants aged ≥ 70 years. Although the overall correlation between age and total MoCA score was moderate and not statistically significant (r = −0.298, *p* = 0.116), these categorical data suggest a clear trend toward lower cognitive performance with advancing age, even in an asymptomatic, comorbidity-free cohort referred for sleep evaluation.

**Fig. 1:**
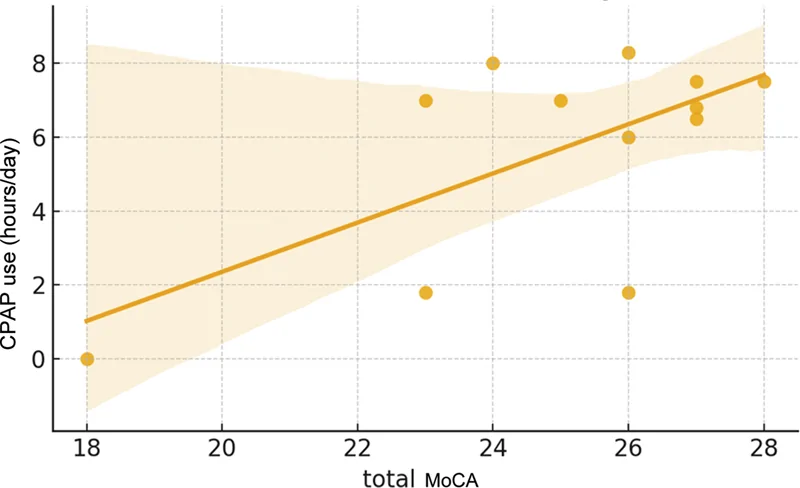
Montreal Cognitive Assessment (MoCA) score versus hours of use of continuous positive airway pressure (CPAP).

Mean MoCA scores were similar between men (25.25) and women (25.08), with no significant difference (t = 0.21, *p* = 0.838).

Notably, 4 of the 7 participants (57.1%) in the AHI 5 to 15 group who would require symptoms for an OSA diagnosis under current guidelines scored below the cognitive impairment cutoff, despite being asymptomatic by history.

Correlation analyses between MoCA performance and CPAP compliance measures revealed that higher global cognitive function was associated with greater adherence. Specifically, total MoCA scores were moderately correlated with average hours of CPAP use per day (r = 0.65, *p* = 0.023) and with the percentage of nights used > 4 hours (r = 0.54, *p* = 0.073). Similarly, the attention subdomain demonstrated moderate correlations with both average daily use (r = 0.61, *p* = 0.026) and nights used > 4 hours (r = 0.57, *p* = 0.044). Additionally, the language domain showed a strong negative correlation with residual AHI (r = −0.69, *p* = 0.019). Other MoCA subdomains, including visuospatial/executive, naming, abstraction, orientation, and delayed recall, were not significantly correlated with any compliance measures. Overall, the observed associations were moderate rather than strong, with global cognition and attention emerging as the most consistent predictors of CPAP adherence.

Figures 1 and 2 further illustrate the relationship between global cognitive performance and CPAP adherence. Figures 1 demonstrates the positive association between total MoCA score and average CPAP use, while Figure 2 shows the distribution of total MoCA scores according to CPAP adherence group (=4 versus <4 hours/day).

**Fig. 2:**
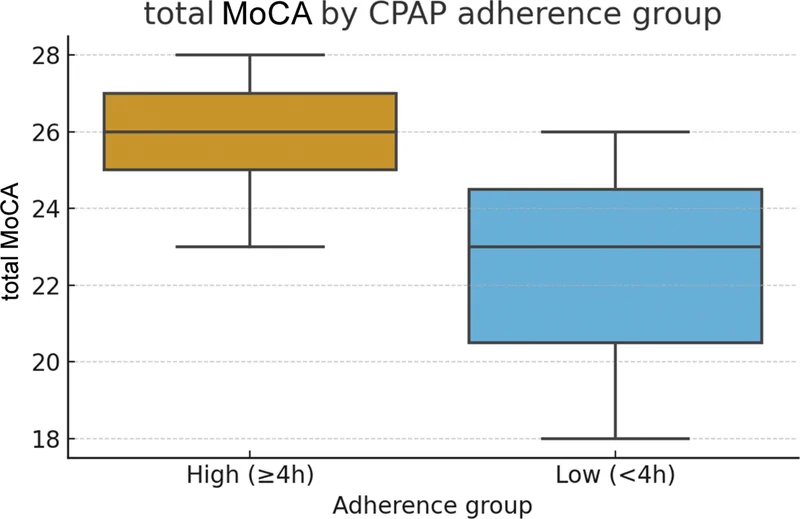
Score on the MoCA according to CPAP use (<4 hours/day and ≥4 hours/day).

## Discussion

Snoring is the most frequent sign of OSA. 27 In this cohort of adults without partial or total impairment of higher mental functions, depression (which could have introduced bias), 28 major comorbidities, or use of medications affecting cognition, we found a substantial proportion with evidence of cognitive impairment on MoCA, despite being asymptomatic according to standard clinical history. Notably, cognitive decline was more frequent with advancing age, 29 with prevalence increasing from 1/3 among participants younger than 60 years to more than 2/3 among those older than 70 years. Although the correlation between age and total MoCA score did not reach statistical significance, the age-stratified results suggest an important trend toward reduced cognitive performance with increasing age in this population.

Although several variables deviated from normality, the consistency in direction and magnitude of associations across both Pearson's and Spearman's analyses supports the robustness of our findings. The observed relationships between cognitive performance and CPAP adherence remained moderate in effect size across analytic approaches, suggesting that these associations are not driven by distributional assumptions but reflect clinically meaningful patterns within the data, despite the modest sample size.

Importantly we also found that higher cognitive performance, as measured by the MoCA, was associated with greater adherence to CPAP therapy. Specifically, total MoCA scores correlated positively with nightly hours of CPAP use, and the Attention subdomain demonstrated consistent associations with both average use and percentage of nights with >4 hours of use. These findings suggest that attentional capacity may play a critical role in patients' ability to adopt and sustain CPAP treatment behaviors. Conversely, lower Language scores were associated with higher residual AHI, which may reflect difficulties in patient–provider communication, device troubleshooting, or comprehension of treatment instructions.

Our results align with prior literature indicating that cognitive deficits, particularly in executive function and attention, are common in patients with OSA and may influence treatment adherence. Importantly, the observed associations emphasize that cognitive screening tools such as the MoCA may help identify patients at risk for suboptimal CPAP use. Incorporating targeted support strategies, such as simplified instructions, enhanced education, or caregiver involvement, could improve adherence in patients with cognitive vulnerabilities.

While our findings highlight clinically meaningful relationships, they should be interpreted in light of the study's sample size and cross-sectional design. Future studies with larger cohorts and longitudinal follow-up are needed to confirm these associations and explore whether cognitive interventions or tailored adherence support can improve CPAP outcomes in this population.

Cognitive dysfunction in OSA is thought to result from a combination of intermittent hypoxia, sleep fragmentation, and neuroinflammatory processes, which may be more pronounced or have greater clinical impact in older adults. Early identification of cognitive decline in patients with mild OSA could provide stronger justification for initiating positive airway pressure (PAP) therapy, particularly in those at risk of progressive cognitive deterioration. 30 Previous studies have demonstrated improvements in attention, executive function, and memory following PAP use, especially when treatment is initiated early.

Importantly, several of these patients were categorized within an AHI range of 5 to 15, suggesting that values below traditionally recognized thresholds of concern may gradually induce vascular irritation with cerebral sequelae, depriving patients of early treatment that could improve prognosis. These findings suggest that incorporating a brief cognitive screening tool, such as the MoCA, may be useful in the evaluation of patients with suspected OSA, though larger prospective studies are needed to confirm this approach. Early identification of cognitive deficits may provide justification for initiating CPAP therapy, which in the long term may improve systemic inflammatory response and cognitive decline. 16 This therapy remains the gold standard for OSA treatment; however, despite its established efficacy, adherence continues to be its primary challenge. 3

Our findings underscore the importance of incorporating brief cognitive tests such as the MoCA, an accessible and easily implemented measure, into the initial evaluation of patients undergoing assessment for OSA, particularly in older individuals or those with borderline AHI values. Furthermore, longitudinal follow-up could serve as a sentinel measure, alerting clinicians and patients to the potential benefit of disease-modifying therapies. 31

It is important to note that factors such as increasing age and lower educational level negatively affect MoCA performance. 32 The authors also consider it valuable to replicate this study with biomarkers capable of detecting early progression of cognitive decline, such as neurofilament light chain (NfL) 33 and Tau protein, 34 in the same population, given that brain parenchymal volumetry and nuclear medicine imaging typically only reflect advanced disease states. 35

## Conclusion

In older adults without impairment of higher mental functions referred for sleep evaluation with PSG, cognitive decline was documented using the MoCA test. This study fulfills its primary objective by demonstrating a high prevalence of cognitive impairment (MoCA < 26) of 51.7% in a cohort of asymptomatic adults undergoing PSG. Notably, 4 of the 7 participants (57.1%) within the AHI range of 5 to 15 events/hour had cognitive impairment despite being asymptomatic by clinical history, a group that would require the presence of symptoms to establish an OSA diagnosis and initiate CPAP therapy, according to current guidelines. These findings suggest that even levels of OSA traditionally considered mild, often underestimated in routine clinical practice, may be associated with clinically relevant neurocognitive alterations, potentially mediated by intermittent hypoxia and vascular dysfunction.

Regarding secondary objective, the most affected cognitive domains were delayed recall, attention, and language, supporting the hypothesis of early involvement of memory and executive related functions in this population. No significant differences in MoCA scores were observed by sex, whereas a trend toward poorer cognitive performance with increasing age was evident, consistent with prior literature.

Overall, these results suggest that brief cognitive screening tools such as the MoCA may help identify patients at risk for suboptimal outcomes, but further prospective studies in larger cohorts are needed before routine implementation can be recommended. Early identification of cognitive impairment may have important clinical implications, especially for subgroups of patients who could benefit from early interventions, such as CPAP therapy, with the potential to modify disease trajectory and mitigate the risk of progressive cognitive decline.

## References

[1] Hofauer B Braumann B Heiser C Diagnosis and treatment of isolated snoring-open questions and areas for future research Sleep Breath 2021 25 02 1011 1017 10.1007/s11325-020-02138-6 PMC8195801 32623557

[2] Huyett P What is snoring? JAMA Otolaryngol Head Neck Surg 2023 149 03 286 10.1001/jamaoto.2022.4615 36701136

[3] Ferreira L GDA Miranda V SG Baseggio M EP Marcolino M AZ Vidor D CGM Myofunctional therapy for the treatment of obstructive sleep apnea: systematic review and meta-analysis Int Arch Otorhinolaryngol 2025 29 01 1 10 10.1055/s-0044-1801780 PMC11793941 39906481

[4] Sowho M Sgambati F Guzman M Schneider H Schwartz A Snoring: a source of noise pollution and sleep apnea predictor Sleep (Basel) 2020 43 06 zsz305 10.1093/sleep/zsz305 PMC8152862 31837267

[5] Dantas ABdA Gonçalves F M Martins A A Worldwide prevalence and associated risk factors of obstructive sleep apnea: a meta-analysis and meta-regression Sleep Breath 2023 27 06 2083 2109 10.1007/s11325-023-02810-7 36971971

[6] Wang W Pang J Zhang Y X Mo Y M Zhang J Wei Z H Clinical effect of a customized adjustable mandibular advancement device in obstructive sleep apnea Br J Hosp Med (Lond) 2024 85 08 1 17 10.12968/hmed.2024.0189 39212566

[7] Iannella G Magliulo G Greco A Obstructive sleep apnea syndrome: from symptoms to treatment Int J Environ Res Public Health 2022 19 04 2459 10.3390/ijerph19042459 PMC8872290 35206645

[8] Destors M Tamisier R Galerneau L M Lévy P Pépin J L [Pathophysiology of obstructive sleep apnea syndrome and its cardiometabolic consequences] Presse Med 2017 46 04 395 403 10.1016/j.lpm.2016.09.008 28126503

[9] Rundo J V Obstructive sleep apnea basics Cleve Clin J Med 2019 86(9, Suppl 1):2 9 10.3949/ccjm.86.s1.02 31509498

[10] Qiu K Mao M Hu Y Gender-specific association between obstructive sleep apnea and cognitive impairment among adults Sleep Med 2022 98 158 166 10.1016/j.sleep.2022.07.004 35870305

[11] Chun C T Seward K Patterson A Melton A MacDonald-Wicks L Evaluation of Available Cognitive Tools Used to Measure Mild Cognitive Decline: A Scoping Review Nutrients 2021 13 11 3974 10.3390/nu13113974 PMC8623828 34836228

[12] Custodio N Montesinos R Lira D Herrera-Pérez E Bardales Y Valeriano-Lorenzo L Mixed dementia: A review of the evidence Dement Neuropsychol 2017 11 04 364 370 10.1590/1980-57642016dn11-040005 PMC5769994 29354216

[13] Ding X Wang J Ji L R [Characteristics of white matter hyperintensities in young and middle-aged male patients with obstructive sleep apnea and its impact on cognitive function] Zhonghua Jie He He Hu Xi Za Zhi 2025 48 04 350 357 10.3760/cma.j.cn112147-20241017-00616 40159053

[14] Peng W Chen R Jiang Z [Correlation between cognitive function and hippocampal atrophy and cerebral white matter lesions in patients with obstructive sleep apnea hypopnea syndrome] Zhonghua Yi Xue Za Zhi 2014 94 10 724 728 24844952

[15] Li Q Li N Z Li T T Sun Y Q Wang J Chen R [The characteristics and determining factors of attention and short-term memory impairment in young patients with obstructive sleep apnea-hypopnea syndrome] Zhonghua Jie He He Hu Xi Za Zhi 2019 42 06 419 425 10.3760/cma.j.issn.1001-0939.2019.06.004 31189227

[16] Sun L Chen R Wang J [Association between inflammation and cognitive function and effects of continuous positive airway pressure treatment in obstructive sleep apnea hypopnea syndrome] Zhonghua Yi Xue Za Zhi 2014 94 44 3483 3487 25622738

[17] Rattanabannakit C Kuendee S Tungwacharapong P Pimolsri C Senanarong V Chotinaiwattarakul W Subjective cognitive complaints and objective cognitive impairment in patients suspected of obstructive sleep apnea who underwent polysomnography Int J Geriatr Psychiatry 2024 39 01 e6055 10.1002/gps.6055 38213266

[18] Berisha D E Rizvi B Chappel-Farley M G Association of obstructive sleep apnea–related hypoxemia with white matter hyperintensities and medial temporal lobe changes in older adults Neurology 2025 104 11 e213639 10.1212/WNL.0000000000213639 PMC12060793 40334140

[19] Zacharias H U Weihs A Habes M Association between obstructive sleep apnea and cerebral white matter hyperintensities in a population-based cohort in Germany JAMA Netw Open 2021 4 10 e2128225 10.1001/jamanetworkopen.2021.28225 PMC8493431 34609493

[20] Chokesuwattanaskul A Chirakalwasan N Jaimchariyatam N Associations between hypoxia parameters in obstructive sleep apnea and cognition, cortical thickness, and white matter integrity in middle-aged and older adults Sleep Breath 2021 25 03 1559 1570 10.1007/s11325-020-02215-w 33057925

[21] Nasreddine Z S Phillips N A Bédirian V The Montreal Cognitive Assessment, MoCA: a brief screening tool for mild cognitive impairment J Am Geriatr Soc 2005 53 04 695 699 10.1111/j.1532-5415.2005.53221.x. Erratum in: J Am Geriatr Soc 2019;67(09):1991. Doi 10.1111/jgs.15925 15817019

[22] Ciesielska N Sokołowski R Mazur E Podhorecka M Polak-Szabela A Kędziora-Kornatowska K Is the Montreal Cognitive Assessment (MoCA) test better suited than the Mini-Mental State Examination (MMSE) in mild cognitive impairment (MCI) detection among people aged over 60? Meta-analysis Psychiatr Pol 2016 50 05 1039 1052 10.12740/PP/45368 27992895

[23] Borland E Nägga K Nilsson P M Minthon L Nilsson E D Palmqvist S The Montreal Cognitive Assessment: normative data from a large Swedish population-based cohort J Alzheimers Dis 2017 59 03 893 901 10.3233/JAD-170203 PMC5545909 28697562

[24] Ogbu I Menon T Chahil V Kahlon A Devanand D Kalra D K Sleep Disordered Breathing and Neurocognitive Disorders J Clin Med 2024 13 17 5001 10.3390/jcm13175001 PMC11396397 39274214

[25] Zhou J Camacho M Tang X Kushida C A A review of neurocognitive function and obstructive sleep apnea with or without daytime sleepiness Sleep Med 2016 23 99 108 10.1016/j.sleep.2016.02.008 27288049

[26] Neves J M Maciel C A Alexandre-Sousa P Benefits of Treating Obstructive Sleep Apnea in Cognition: Systematic Literature Review J Dement Alzheimer's Dis 2025 2 01 3 10.3390/jdad2010003

[27] Chiang J K Lin Y C Lu C M Kao Y H Correlation between snoring sounds and obstructive sleep apnea in adults: a meta-regression analysis Sleep Sci 2022 15 04 463 470 10.5935/1984-0063.20220068 PMC9670768 36419807

[28] Blair M Coleman K Jesso S Depressive Symptoms Negatively Impact Montreal Cognitive Assessment Performance: A Memory Clinic Experience Can J Neurol Sci 2016 43 04 513 517 10.1017/cjn.2015.399 26842678

[29] Marchi N A Solelhac G Berger M Obstructive sleep apnoea and 5-year cognitive decline in the elderly Eur Respir J 2023 61 04 2.201621E6 10.1183/13993003.01621-2022 PMC10133583 36796834

[30] Gagnon K Baril A A Gagnon J F Cognitive impairment in obstructive sleep apnea Pathol Biol (Paris) 2014 62 05 233 240 10.1016/j.patbio.2014.05.015 25070768

[31] Thawabteh A M Ghanem A W AbuMadi S Recent Advances in Therapeutics for the Treatment of Alzheimer's Disease Molecules 2024 29 21 5131 10.3390/molecules29215131 PMC11547905 39519769

[32] Freitas S Simões M R Alves L Santana I Montreal Cognitive Assessment: influence of sociodemographic and health variables Arch Clin Neuropsychol 2012 27 02 165 175 10.1093/arclin/acr116 22277128

[33] Lim B Grøntvedt G R Bathala P CSF neurofilament light may predict progression from amnestic mild cognitive impairment to Alzheimer's disease dementia Neurobiol Aging 2021 107 78 85 10.1016/j.neurobiolaging.2021.07.013 34403936

[34] Suárez-Calvet M CSF p-tau231: A biomarker for early preclinical Alzheimer? EBioMedicine 2022 77 103936 10.1016/j.ebiom.2022.103936 PMC8919211 35290829

[35] Miller-Thomas M M Sipe A L Benzinger T L McConathy J Connolly S Schwetye K E Multimodality Review of Amyloid-related Diseases of the Central Nervous System Radiographics 2016 36 04 1147 1163 10.1148/rg.201615017 PMC4976469 27399239

